# Possible Increase in Serum FABP4 Level Despite Adiposity Reduction by Canagliflozin, an SGLT2 Inhibitor

**DOI:** 10.1371/journal.pone.0154482

**Published:** 2016-04-28

**Authors:** Masato Furuhashi, Megumi Matsumoto, Shinya Hiramitsu, Akina Omori, Marenao Tanaka, Norihito Moniwa, Hideaki Yoshida, Junnichi Ishii, Tetsuji Miura

**Affiliations:** 1 Department of Cardiovascular, Renal and Metabolic Medicine, Sapporo Medical University School of Medicine, S-1, W-16, Chuo-ku, Sapporo, 060-8543, Japan; 2 Hiramitsu Heart Clinic, Shiroshita-cho 2-35, Minami-ku, Nagoya, 457-0047, Aichi, Japan; 3 Department of Joint Research Laboratory of Clinical Medicine, Fujita Health University School of Medicine, Toyoake, Aichi, Japan; Chiba University Graduate School of Medicine, JAPAN

## Abstract

**Background:**

Fatty acid-binding protein 4 (FABP4/A-FABP/aP2) is secreted from adipocytes in association with catecholamine-induced lipolysis, and elevated serum FABP4 level is associated with obesity, insulin resistance and atherosclerosis. Secreted FABP4 as a novel adipokine leads to insulin resistance via increased hepatic glucose production (HGP). Sodium-glucose cotransporter 2 (SGLT2) inhibitors decrease blood glucose level via increased urinary glucose excretion, though HGP is enhanced. Here we investigated whether canagliflozin, an SGLT2 inhibitor, modulates serum FABP4 level.

**Methods:**

Canagliflozin (100 mg/day) was administered to type 2 diabetic patients (n = 39) for 12 weeks. Serum FABP4 level was measured before and after treatment.

**Results:**

At baseline, serum FABP4 level was correlated with adiposity, renal dysfunction and noradrenaline level. Treatment with canagliflozin significantly decreased adiposity and levels of fasting glucose and HbA1c but increased average serum FABP4 level by 10.3% (18.0 ± 1.0 vs. 19.8 ± 1.2 ng/ml, P = 0.008), though elevation of FABP4 level after treatment was observed in 26 (66.7%) out of 39 patients. Change in FABP4 level was positively correlated with change in levels of fasting glucose (r = 0.329, P = 0.044), HbA1c (r = 0.329, P = 0.044) and noradrenaline (r = 0.329, P = 0.041) but was not significantly correlated with change in adiposity or other variables.

**Conclusions:**

Canagliflozin paradoxically increases serum FABP4 level in some diabetic patients despite amelioration of glucose metabolism and adiposity reduction, possibly via induction of catecholamine-induced lipolysis in adipocytes. Increased FABP4 level by canagliflozin may undermine the improvement of glucose metabolism and might be a possible mechanism of increased HGP by inhibition of SGLT2.

**Trial Registration:**

UMIN-CTR Clinical Trial UMIN000018151

## Introduction

Fatty acid-binding proteins (FABPs), a family of intracellular lipid chaperones, are about 14-15-kDa predominantly cytosolic proteins that can reversibly bind hydrophobic ligands, such as saturated and unsaturated long-chain fatty acids [1–3]. FABPs have been proposed to facilitate the transport of lipids to specific compartments in the cell [1]. Among FABPs, fatty acid-binding protein 4 (FABP4), also referred to as adipocyte FABP (A-FABP) or aP2, is mainly expressed in both adipocytes and macrophages and plays an important role in the development of obesity, insulin resistance, type 2 diabetes mellitus and atherosclerosis [4–6]. We previously demonstrated that the use of a small molecule FABP4-specific inhibitor might be a novel therapeutic strategy against insulin resistance, type 2 diabetes mellitus and atherosclerosis [7].

Recently, FABP4 has been reported to be secreted from adipocytes in association with lipolysis via a non-classical secretion pathway [8–11], though there are no typical secretory signal peptides in the sequence of FABP4 [1]. Previous studies using *in vitro* and *in vivo* experiments showed that FABP4 acts as an adipokine leading to the development of hepatic insulin resistance through increased hepatic glucose production [9] and atherosclerosis [12]. It has also been reported that elevated serum FABP4 concentration is associated with obesity, insulin resistance, type 2 diabetes mellitus, hypertension, cardiac dysfunction, renal dysfunction, dyslipidemia, atherosclerosis and cardiovascular events [8, 13–23]. However, little is known about the modulation of serum FABP4 level by anti-diabetic agents except for thiazolidinedione [24] and a dipeptidyl peptidase-4 (DPP-4) inhibitor [25].

For treatment of type 2 diabetes mellitus, sodium-glucose cotransporter 2 (SGLT2) inhibitors have recently become available. SGLT2 inhibitors decrease blood glucose level through increased glucose excretion in urine [26]. On the other hand, SGLT2 inhibitors have been reported to enhance hepatic glucose production [27, 28] by an unknown molecular mechanism. In the present study, we investigated the impact of SGLT2 inhibitor therapy on serum FABP4 level in patients with type 2 diabetes mellitus.

## Materials and Methods

This study registered in UMIN-CTR Clinical Trial (UMIN000018151) conformed to the principles outlined in the Declaration of Helsinki and was performed with the approval of the Ethical Committee of Fujita Health University. Written informed consent was received from all of the study subjects. The protocol for this trial and supporting TREND checklist are available as S1 checklist and S2 protocol.

### Study subjects

Patients with type 2 diabetes mellitus were consecutively recruited from outpatient clinics affiliated with Fujita Health University from October 2014 through March 2015. Exclusion criteria were findings of serious co-morbidities such as hepatic, cerebrovascular, cardiovascular or renal disease. Patients treated with thiazolidinediones, peroxisome proliferator-activated receptor γ (PPARγ) agonists, were also excluded since expression and serum level of FABP4 as a target gene has been reported to be directly regulated by PPARγ activation [1, 24]. The primary endpoint was assessment of change in level of Hemoglobin A1c (HbA1c). The secondary endpoint was assessment of changes in several glucose metabolism-related parameters, including adiposity and levels of fasting glucose, insulin and FABP4. Samples of blood and urine were collected before and after treatment with canagliflozin (100 mg/day), an SGLT2 inhibitor, for 12 weeks. For blood sampling, patients were kept in the supine position for 20 min after an overnight fast. Samples of plasma, serum and urine were analyzed immediately or stored at -80°C until biochemical analyses.

### Measurements

The serum concentration of FABP4 was measured using a commercially available enzyme-linked immunosorbent assay kit (Biovendor R&D, Modrice, Czech Republic). The accuracy, precision and reproducibility of the kit have been described previously [8]. The intra- and inter-assay coefficient variances in the kits were < 5%. Serum high molecular weight (HMW)-adiponectin level was measured using a commercially available enzyme-linked immunosorbent assay kit (Fujirebio Inc., Tokyo, Japan). Fasting plasma glucose was determined by the glucose oxidase method. Fasting plasma insulin was measured by a chemiluminescent enzyme immunoassay method. HbA1c was determined by a latex coagulation method and was expressed in National Glycohemoglobin Standardization Program (NGSP) scale. Creatinine (Cr), blood urea nitrogen (BUN), uric acid, aspartate transaminase (AST), alanine aminotransferase (ALT), γ-glutamyl transpeptidase (γ-GTP) and lipid profiles, including total cholesterol, high-density lipoprotein (HDL) cholesterol and triglycerides, were determined by enzymatic methods. Low-density lipoprotein (LDL) cholesterol level was calculated by the Friedewald equation. Cystatin C (Cys-C) was determined by a latex coagulation method. Brain natriuretic peptide (BNP) was measured using an assay kit (Shionogi & Co., Osaka, Japan). High-sensitivity C-reactive protein (hsCRP) was measured by a nephelometry method. Plasma levels of adrenaline and noradrenaline were measured by high-performance liquid chromatography. Homeostasis model assessment of insulin resistance (HOMA-R) was calculated by the previously reported formula: HOMA-R = insulin (μU/ml) × glucose (mg/dl) / 405. As an index of renal function, estimated glomerular filtration rate (eGFR) was calculated by an equation for Japanese [29]: eGFR (ml/min/1.73m^2^) = 194 × Cr^(-1.094)^ × age^(-0.287)^ × 0.739 (if female). Urinary albumin-to-creatinine ratio (UACR; mg/gCr) was used as a marker of microalbuminuria. Body mass index (BMI) was calculated as body weight (in kilograms) divided by the square of body height (in meters).

### Statistical analysis

The sample size was calculated on the basis of assumption that the difference between HbA1c levels before and after treatment with canagliflozin for 12 weeks would be 0.7% and that the standard deviations in HbA1c level at baseline and 12 weeks would be 1.2% and 1.2%, respectively. Correlation coefficient of HbA1c levels at baseline and 12 weeks would be 0.50. To detect such a significant difference before and after treatment in the statistical situation of a power greater than 90% with a two-sided type 1 error rate of 0.05, at least 33 patients were required.

Numeric variables are expressed as means ± SEM. The distribution of each parameter was tested for its normality using the Shapiro-Wilk W test, and non-normally distributed parameters were logarithmically transformed for regression analyses. The correlation between two variables was evaluated using Pearson’s correlation coefficient. Comparison between two groups was done with the chi-square test, Wilcoxon signed-rank test for paired samples and Mann-Whitney's U test for unpaired samples. Stepwise regression analysis was performed to identify independent determinants of FABP4 concentration using age, gender and the variables with a significant correlation in simple regression analysis as independent predictors in a forward direction with F value ≥ 4 for the entry, and a subsequent multiple regression analysis was done to show the t-ratio calculated as the ratio of regression coefficient and standard error of regression coefficient and the percentage of variance in the FABP4 concentration that the selected independent predictors explained (R^2^). A p value of less than 0.05 was considered statistically significant. All data were analyzed by using JMP 9 for Macintosh (SAS Institute, Cary, NC).

## Results

**Fig 1:**
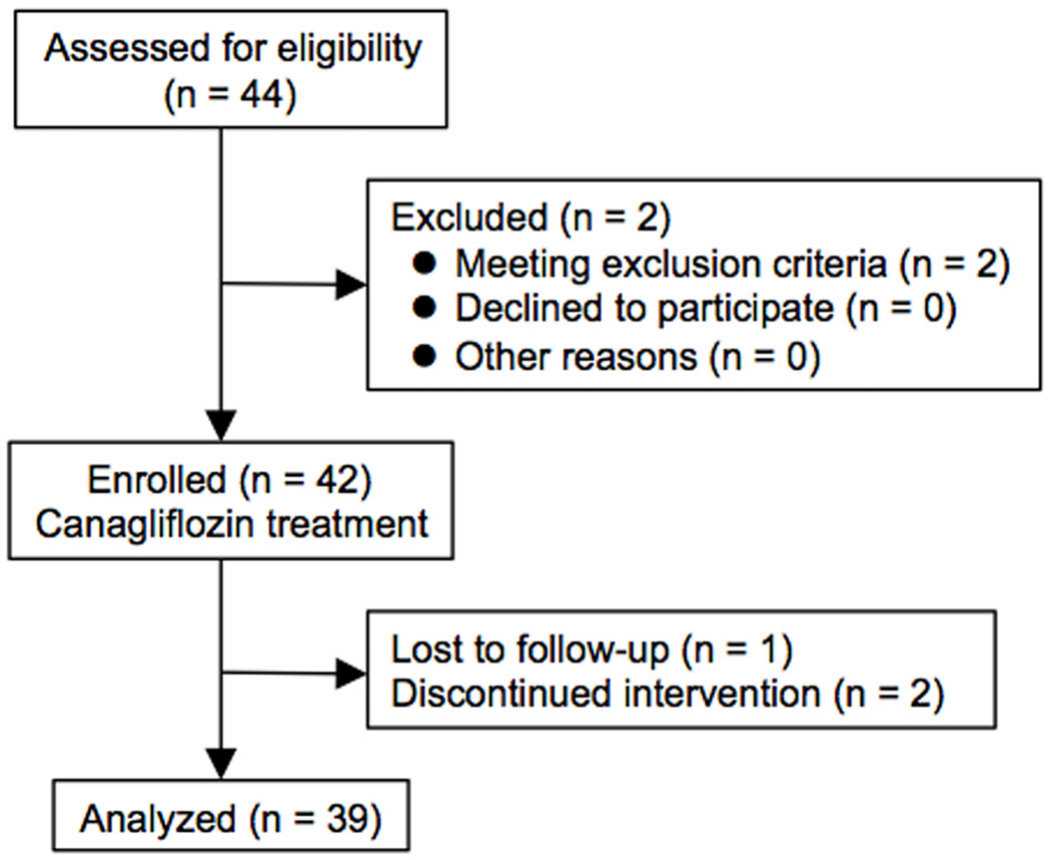
Flow chart of study participants. A total of 44 patients with type 2 diabetes mellitus were recruited, and 39 patients were finally analyzed in the present study.

A patient flow diagram in the present study is shown in Fig 1. A total of 44 patients with type 2 diabetes mellitus were consecutively recruited for the present study. Two patients met the exclusion criteria prior to this study. Hence, 42 patients were enrolled for canagliflozin treatment. Three patients were excluded because of lost to follow-up (n = 1) and discontinued intervention (n = 2). Finally, a total of 39 type 2 diabetic patients (male/female: 28/11) were analyzed in the present study.

Characteristics of the patients are shown in Table 1. Mean age, BMI and waist circumference of the recruited patients were 63.0 ± 1.5 years, 27.9 ± 0.7 kg/m^2^ and 94.4 ± 1.5 cm, respectively. More than 90% of the patients had hypertension and dyslipidemia, and most of the patients had received pharmacological agents for hypertension and dyslipidemia including statins, ezetimibe and fibrates. Medication for type 2 diabetes mellitus included biguanides (17.9%), DPP-4 inhibitors (46.2%) and sulfonylureas (5.1%).

**Table 1:** Background of the patients.

n (M/F)	39 (28/11)
Age (years)	63.0 ± 1.5
Body mass index (kg/m^2^)	27.9 ± 0.7
Waist circumference (cm)	94.4 ± 1.5
Diagnosis	
Hypertension	37 (94.9)
Dyslipidemia	38 (97.4)
Medication	
Biguanides	7 (17.9)
Dipeptidyl peptidase-4 inhibitors	18 (46.2)
Sulfonylureas	2 (5.1)
Angiotensin II receptor blockers	29 (74.4)
Angiotensin-converting enzyme inhibitors	1 (2.6)
Direct renin inhibitor	3 (7.7)
Calcium channel blockers	18 (46.2)
α blockers	1 (2.6)
β blockers	15 (38.5)
Diuretics	0 (0)
Mineralocorticoid receptor antagonists	2 (5.1)
Statins	17 (43.6)
Ezetimibe	14 (35.9)
Fibrates	6 (15.4)

Variables are expressed as n (%) or means ± SEM.

At baseline, serum FABP4 level was positively correlated with BMI, waist circumference, pulse rate and levels of Cys-C and noradrenaline and was negatively correlated with levels of eGFR and hematocrit (Table 2). Stepwise regression analysis using age, gender and the correlated parameters revealed that gender (F = 7.97), BMI (F = 11.94) and Cys-C (F = 15.55) were independent predictors of FABP4 concentration. A subsequent multiple regression analysis showed that gender (male; t = -2.86, P = 0.007), BMI (t = 3.65, P = 0.001) and Cys-C (t = 4.01, P < 0.001) were independently correlated with FABP4 level, explaining a total of 54.9% of the variance in this measure (R^2^ = 0.549) (Table 3), as previously reported [15, 21].

**Table 2:** Simple regression analysis for log FABP4 at baseline.

	r	p
Age	0.150	0.363
Body mass index	0.450	0.004
Waist circumference	0.443	0.005
Systolic blood pressure	0.151	0.359
Diastolic blood pressure	-0.151	0.359
Pulse rate	0.370	0.020
Biochemical data		
Total cholesterol	0.060	0.715
LDL cholesterol	0.111	0.500
HDL cholesterol	-0.079	0.635
log Triglycerides	0.045	0.787
log Fasting glucose	0.120	0.466
HbA1c	0.218	0.182
log Insulin	0.168	0.315
log HOMA-R	0.201	0.225
Blood urea nitrogen	-0.015	0.926
Creatinine	0.187	0.254
eGFR	-0.329	0.044
Cystatin C	0.493	0.002
log UACR	0.235	0.150
Uric acid	0.025	0.882
Hematocrit	-0.365	0.023
log Aspartate transaminase	0.005	0.978
log Alanine transaminase	-0.052	0.753
log γ-glutamyl transpeptidase	-0.186	0.256
log Brain natriuretic peptide	0.200	0.230
log hsCRP	0.162	0.344
log Adrenaline	0.089	0.588
log Noradrenaline	0.329	0.041
log HMW-Adiponectin	0.167	0.311

eGFR, estimated glomerular filtration rate. UACR, urine albumin-to-creatinine ratio; hsCRP, high-sensitivity C-reactive protein; HMW, high molecular weight.

**Table 3:** Multiple regression analysis for log FABP4 at baseline.

	log FABP4	
	t	P
Age	0.47	0.641
Gender (Male)	-2.86	0.007
Body mass index	3.65	0.001
Cystatin C	4.01	<0.001

R^2^ = 0.549

**Fig 2:**
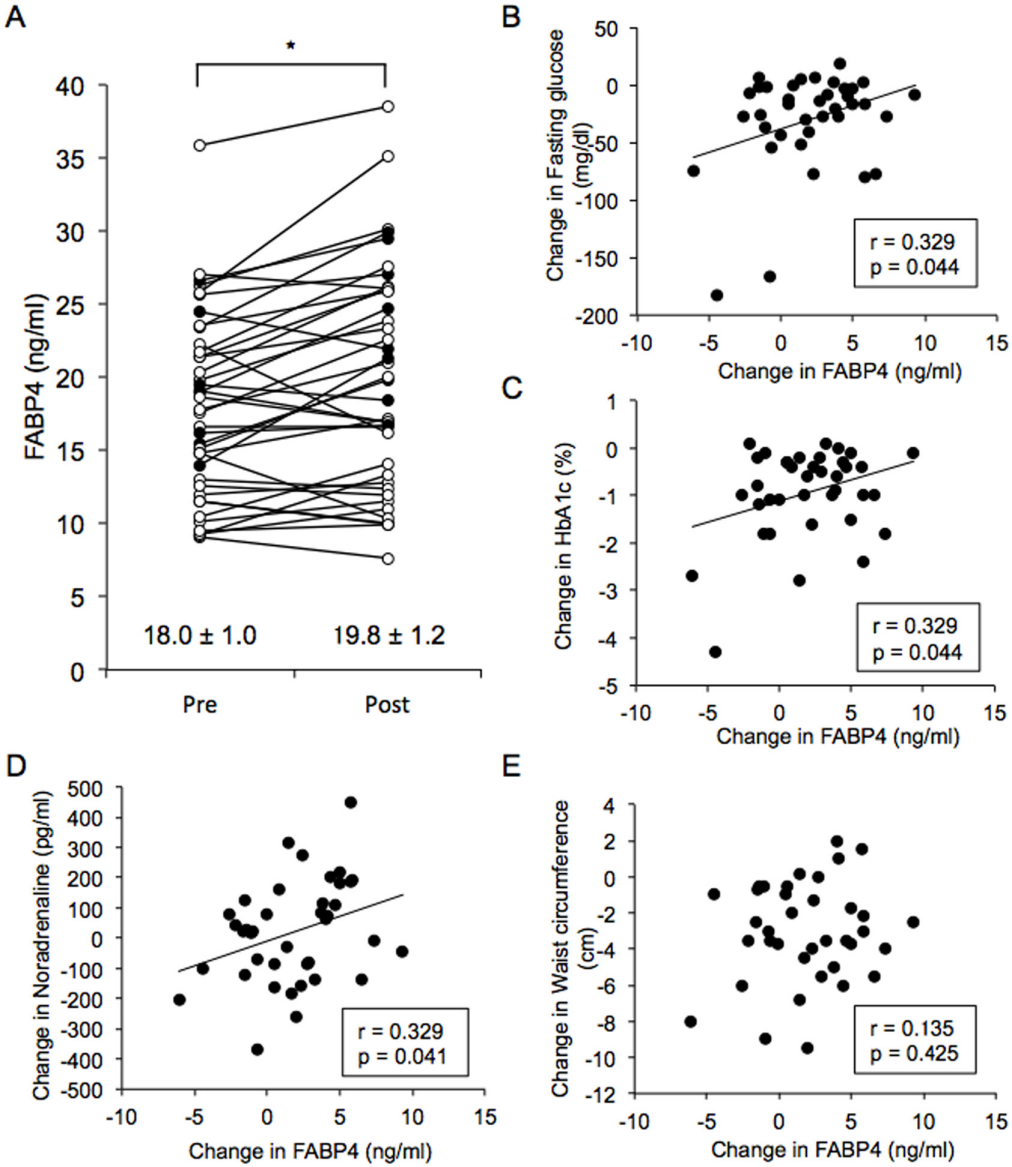
Effect of canagliflozin on FABP4 level. **A.** Treatment with canagliflozin (100 mg/day) for 12 weeks significantly increased FABP4 level in patients with type 2 diabetes mellitus (n = 39; male/female: 28/11). Open circles: males, closed circles: females. *P = 0.008. **B-E.** Change in FABP4 level was positively correlated with changes in levels of fasting glucose (B), HbA1c (C) and noradrenaline (D) but was not significantly correlated with change in waist circumference (E).

Treatment with canagliflozin for 12 weeks significantly decreased BMI, waist circumference, systolic and diastolic blood pressures, pulse rate and levels of fasting glucose, insulin, HOMA-R, HbA1c, uric acid, ALT, γ-GPT and hsCRP, while it oppositely increased levels of BUN, Cys-C, hematocrit and HMW-adiponectin (Table 4). No significant change after canagliflozin treatment was found in levels of total cholesterol, HDL cholesterol, LDL cholesterol, triglycerides, Cr, eGFR, UACR, AST, BNP, adrenaline and noradrenaline. Interestingly, treatment with canagliflozin significantly increased the mean value of serum FABP4 concentration by 10.3% (18.0 ± 1.0 vs. 19.8 ± 1.2 ng/ml, P = 0.008) (Fig 2A) despite reduction of adiposity. However, the direction and magnitude of change in FABP4 level were different on a patient-to-patient basis, and FABP4 level was increased and decreased in 66.7% (26/39) and 33.3% (13/39) of the patients, respectively. There was no significant difference in baseline clinical parameters between the two groups of patients showing different responses of FABP4 level to canagliflozin treatment (Table 5). Reduction of HOMA-R by canagliflozin was significantly smaller in patients with increased FABP4 after canagliflozin treatment than in those with decreased FABP4 (-0.53 ± 0.28 vs. -2.02 ± 0.43, P = 0.009).

**Table 4:** Characteristics of the patients treated with canagliflozin for 12 w.

	Pre	Post	P
Body mass index (kg/m^2^)	27.9 ± 0.7	26.5 ± 0.7*	<0.001
Waist circumference (cm)	94.4 ± 1.5	90.9 ± 1.5*	<0.001
Systolic blood pressure (mmHg)	138.0 ± 0.8	128.1 ± 0.6*	<0.001
Diastolic blood pressure (mmHg)	84.1 ± 0.4	74.9 ± 0.6*	<0.001
Pulse rate (beats/min)	73.2 ± 0.5	70.9 ± 0.9*	0.003
Biochemical data			
Fasting glucose (mg/dl)	145.9 ± 6.9	115.8 ± 3.0*	<0.001
Insulin (μU/ml)	9.7 ± 0.9	8.0 ± 0.7*	0.006
HOMA-R	3.36 ± 0.34	2.34 ± 0.23*	<0.001
HbA1c (%)	7.4 ± 0.2	6.5 ± 0.1*	<0.001
Total cholesterol (mg/dl)	179.9 ± 4.7	185.4 ± 5.3	0.306
HDL cholesterol (mg/dl)	50.0 ± 1.9	52.8 ± 2.4	0.105
LDL cholesterol (mg/dl)	103.7 ± 4.1	107.5 ± 4.8	0.316
Triglycerides (mg/dl)	129.5 ± 9.5	127.8 ± 8.0	0.673
Blood urea nitrogen (mg/dl)	14.7 ± 0.6	16.5 ± 0.6*	0.026
Creatinine (mg/dl)	0.73 ± 0.03	0.74 ± 0.03	0.680
eGFR (ml/min/1.73m^2^)	80.4 ± 2.9	80.5 ± 3.0	0.658
Cystatin C (mg/l)	0.99 ± 0.04	1.04 ± 0.04*	0.001
UACR (mg/gCr)	33.8 ± 10.1	30.8 ± 7.4	0.863
Uric acid (mg/dl)	5.0 ± 0.2	4.4 ± 0.1*	<0.001
Hematocrit (%)	44.5 ± 0.5	47.1 ± 0.6*	<0.001
Aspartate transaminase (U/l)	29.8 ± 2.5	24.9 ± 1.4	0.287
Alanine transaminase (U/l)	36.7 ± 4.1	25.9 ± 1.8*	0.001
γ-glutamyl transpeptidase (U/l)	56.4 ± 8.9	40.8 ± 6.0	<0.001
Brain natriuretic peptide (pg/ml)	26.2 ± 4.9	24.4 ± 4.5	0.304
hsCRP (mg/dl)	0.15 ± 0.02	0.11 ± 0.02*	0.019
Adrenaline (pg/ml)	25.1 ± 2.8	25.3 ± 2.5	0.730
Noradrenaline (pg/ml)	407.4 ± 30.6	428.0 ± 31.0	0.502
HMW-adiponectin (μg/ml)	2.7 ± 0.3	3.1 ± 0.3*	<0.001
FABP4 (ng/ml)	18.0 ± 1.0	19.8 ± 1.2*	0.008

Variables are expressed as means ± SEM.

*P < 0.05 vs. Pre.

eGFR, estimated glomerular filtration rate; UACR, urine albumin-to-creatinine ratio; hsCRP, high-sensitivity C-reactive protein; HMW, high molecular weight.

**Table 5:** Characteristics of the patients with decreased and increased FABP4 level by canagliflozin.

	FABP4 level		
	Down	Up	P
n (M/F)	13 (10/3)	26 (18/8)	0.719
Body mass index (kg/m^2^)	27.8 ± 0.9	27.9 ± 0.9	0.896
Waist circumference (cm)	95.3 ± 2.5	94.0 ± 2.0	0.690
Systolic blood pressure (mmHg)	136.4 ± 1.1	138.8 ± 1.1	0.148
Diastolic blood pressure (mmHg)	84.5 ± 0.6	83.9 ± 0.6	0.541
Pulse rate (beats/min)	72.0 ± 1.0	73.8 ± 0.5	0.131
Biochemical data			
Fasting glucose (mg/dl)	162.1 ± 15.2	137.8 ± 6.8	0.162
Insulin (μU/ml)	11.6 ± 1.7	8.7 ± 1.1	0.171
HOMA-R	4.32 ± 0.63	2.86 ± 0.38	0.060
HbA1c (%)	7.6 ± 0.4	7.3 ± 0.2	0.485
Total cholesterol (mg/dl)	190.0 ± 10.3	174.8 ± 4.6	0.198
HDL cholesterol (mg/dl)	49.3 ± 3.5	50.3 ± 2.3	0.809
LDL cholesterol (mg/dl)	110.2 ± 7.9	100.5 ± 4.8	0.307
Triglycerides (mg/dl)	131.4 ± 8.9	128.6 ± 13.7	0.864
Blood urea nitrogen (mg/dl)	14.4 ± 1.1	14.9 ± 0.7	0.706
Creatinine (mg/dl)	0.69 ± 0.05	0.75 ± 0.04	0.369
eGFR (ml/min/1.73m^2^)	87.3 ± 4.5	77.5 ± 3.8	0.115
Cystatin C (mg/l)	0.91 ± 0.06	1.03 ± 0.05	0.123
UACR (mg/gCr)	18.3 ± 5.6	41.5 ± 14.8	0.152
Uric acid (mg/dl)	4.7 ± 0.2	5.1 ± 0.3	0.272
Hematocrit (%)	46.1 ± 0.9	43.8 ± 0.6	0.051
Aspartate transaminase (U/l)	32.6 ± 5.0	28.4 ± 2.8	0.470
Alanine transaminase (U/l)	42.8 ± 7.4	33.6 ± 4.9	0.310
γ-glutamyl transpeptidase (U/l)	72.2 ± 19.0	48.5 ± 9.3	0.276
Brain natriuretic peptide (pg/ml)	11.2 ± 3.7	14.0 ± 6.8	0.530
hsCRP (mg/dl)	0.13 ± 0.03	0.16 ± 0.04	0.602
Adrenaline (pg/ml)	19.2 ± 2.8	18.1 ± 3.9	0.070
Noradrenaline (pg/ml)	353.9 ± 37.2	434.2 ± 41.5	0.159
HMW-adiponectin (μg/ml)	2.2 ± 0.5	3.0 ± 0.4	0.206
FABP4 (ng/ml)	16.9 ± 1.5	18.5 ± 1.3	0.430

Variables are expressed as means ± SEM.

eGFR, estimated glomerular filtration rate; UACR, urine albumin-to-creatinine ratio; hsCRP, high-sensitivity C-reactive protein; HMW, high molecular weight.

Change (Post—Pre) in FABP4 level after canagliflozin treatment was positively correlated with changes in levels of fasting glucose (r = 0.329, P = 0.044) (Fig 2B) and HbA1c (r = 0.329, P = 0.044) (Fig 2C), indicating that improvement in fasting glucose and HbA1c by canagliflozin was less in patients with greater increase in FABP4 level. Furthermore, change in FABP4 level was positively correlated with change in level of noradrenaline (r = 0.329, P = 0.041) (Fig 2D) but was not significantly correlated with change in BMI (r = -0.021, P = 0.901), waist circumference (r = 0.135, P = 0.425) (Fig 2E) or other variables.

## Discussion

The present study showed for the first time that canagliflozin, an SGLT2 inhibitor, increased the average serum FABP4 concentration in patients with type 2 diabetes mellitus despite amelioration of glucose metabolism and reduction of adiposity (Fig 2A, Table 4). However, the direction and magnitude of change in FABP4 level after canagliflozin treatment were different between patients. Compared to patients with decreased FABP4 level by canagliflozin (33.3%), patients with increased FABP4 (66.7%) had significantly smaller improvement of insulin resistance assessed by change in HOMA-R. Furthermore, change in FABP4 level caused by canagliflozin was positively correlated with change in levels of fasting glucose and HbA1c but was not significantly correlated with change in adiposity (Fig 2B, 2C and 2E). These findings suggest that the paradoxical increase in FABP4 concentration by inhibition of SGLT2 is independent of alteration of adiposity and undermines the improvement of glucose metabolism since circulating FABP4 leads to hepatic insulin resistance [9].

It has been reported that SGLT2 inhibitors increase hepatic glucose production [27, 28], though the molecular mechanism has not been completely elucidated. Involvement of various factors has been postulated for the rise in hepatic glucose production: compensatory response by change in glucose level and decrease in insulin secretion [26], increase in glucagon secretion from pancreatic α cells [30] and influx of free fatty acids via lipolysis from adipocytes by attenuated anti-lipolytic action of insulin [31]. It has been shown that FABP4 is secreted from adipocytes in association with lipolysis [9–11]. In the present study, FABP4 concentration at baseline was positively correlated with level of noradrenaline, an index of activation of the sympathetic nerve system (Table 2). Furthermore, change in FABP4 concentration by canagliflozin was positively correlated with change in noradrenaline level (Fig 2D), though there was no significant difference between noradrenalin level before and after treatment with canagliflozin (Table 4). These findings support the notion that enhancement of catecholamine-induced lipolysis in adipocytes by canagliflozin in some patients with type 2 diabetes mellitus contributes to elevation of FABP4 level. Increased FABP4 level by canagliflozin might be a possible mechanism of increased hepatic glucose production by inhibition of SGLT2. To prove this hypothesis, animal experiments in which FABP4-deficient mice are treated with SGLT2 inhibitors will be necessary.

Serum FABP4 level has been reported to predict long-term cardiovascular events [21–23]. The association of cardiovascular events and serum FABP4 level has been explained by a significant role of FABP4 in insulin resistance and atherosclerosis [4–6]. The present study showed that an SGLT2 inhibitor, canagliflozin, increased the average FABP4 level by 10.3% in patients with type 2 diabetes mellitus, though FABP4 level was reduced in 33.3% of the studied patients. The findings suggest that SGLT2 inhibitors might have an adverse effect on cardiovascular events in some diabetic patients. Recently, a large-scale randomized controlled trial study, EMPA-REG OUTCOME [32], showed that empagliflozin, an SGLT2 inhibitor, decreased composite cardiovascular events, mainly due to reduction of heart failure by a hemodynamic effect including osmotic diuresis rather than reduction of blood pressure [33]. However, the incidence of acute myocardial infarction or stroke was not significantly reduced by empagliflozin in the EMPA-REG OUTCOME study [32]. Hence, there is the possibility that the benefit of glycemic control by an SGLT2 inhibitor for atherosclerotic events is partly offset by increased serum FABP4 level. This possibility needs to be further investigated by large-scale clinical studies.

The present study has several limitations. First, the number of patients enrolled was small, and the possibility of type 1 or type 2 errors in statistical tests cannot be excluded. Second, most of the study subjects had been treated at baseline with several drugs, including angiotensin II receptor blockers [34, 35], antidyslipidemic drugs [36, 37] and DPP-4 inhibitors [25], that have been reported to affect circulating FABP4 concentration. Therefore, such drugs might have modulated the change in FABP4 level. Third, adiposity was assessed by BMI and waist circumference but not by computer technologies. Fourth, levels of free fatty acid and glucagon, which are related to lipolysis and hepatic glucose production, were not measured in the present study due to a lack of remaining blood samples. Lastly, the present study lacked a placebo control group. Hence, the difference in FABP4 level before and after canagliflozin treatment may not be totally attributable to this SGLT2 inhibitor. A prospective and placebo-controlled study with larger numbers of subjects is necessary for confirming the impact of SGLT2 inhibitor treatment on circulating FABP4 level and for determining the clinical outcome of treatment with an SGLT2 inhibitor.

In conclusion, treatment with canagliflozin paradoxically increases serum FABP4 level in some patients with type 2 diabetes mellitus despite amelioration of glucose metabolism and reduction of adiposity, and this effect is possibly mediated by catecholamine-induced lipolysis in adipocytes. Increased FABP4 level by canagliflozin may partly offset the benefit of improvement in glucose metabolism and might be a possible mechanism of increased hepatic glucose production by inhibition of SGLT2. A further understanding of the mechanisms of FABP4 expression in and secretion from adipocytes and their pharmacological modulation may enable development of new therapeutic strategies for cardiovascular and metabolic diseases.

## Supporting Information

S1 Checklist TREND checklist of this trial. (PDF)

S1 Protocol This protocol was used in this trial. (PDF)

S2 Protocol English version of this protocol. (DOCX)
