# Supplementary material for: Possible Increase in Serum FABP4 Level Despite Adiposity Reduction by Canagliflozin, an SGLT2 Inhibitor
Source: PLoS One. 2016 Apr 28;11(4):e0154482. doi: 10.1371/journal.pone.0154482 (PMC4849662; doi:10.1371/journal.pone.0154482)
Supplement: S1 Protocol — (PDF) [file pone.0154482.s002.pdf]

2型糖尿病患者におけるカナグリフロジンの生活習慣病パラメータおよび心・腎・血管障害関連因子に及ぼす影響の検討

試験計画書

研究責任者　：石井　潤一
：藤田保健衛生大学　医学部　臨床検査科
：愛知県豊明市沓掛町田楽ヶ窪１－９８

計画書作成日：2014年　9月　10日（第1.0版）
2014年　10月　7日（第1.1版）

目　次

Ⅰ. 研究計画の要約 2
Ⅱ. 研究計画の経緯 2
Ⅲ. 研究計画 2
1. 目的 2
2. 対象 3
3. 同意 3
4. 被験薬 4
5. 試験方法 4
6. 検査項目 5
7. 評価 5
8. 有害事象が発現した場合の処置 5
9. 中止・脱落 6
10. 実施事項における倫理的配慮 6
11. 試験実施計画書の変更等に関する手順 8
12. 試験実施計画書の不遵守発生時の処置 8
13. 目標症例数 8
14. 実施期間 8
15. 本臨床研究の登録.. 9
16. データ解析 9
17. 実施代表者／事務局 9
18. 試験実施施設 9
19. 検査測定機関 10

Ⅰ．研究計画の要約

血糖コントロールが不十分である肥満・高血圧合併2型糖尿病患者を対象に、ナトリウム・グルコース共輸送体2(SGLT2)阻害剤であるカナグリフロジンの生活習慣病パラメータや心・腎・血管障害関連因子への有効性を検討する。

Ⅱ．研究計画の経緯

従来、日本人の糖尿病はインスリン分泌低下型が主要であり、痩せ型の多いことが特徴とされてきた。しかし、食事の欧米化や運動不足等が原因で日本においても肥満の割合は増加しており、それに伴って、糖尿病患者でも肥満者の割合が増えてきている[1)]。肥満は高血圧、脂質異常症の引き金にもなり、2型糖尿病患者にとっては心血管イベントリスクの重積を招きやすくなる。したがって、肥満合併2型糖尿病患者では、血糖値とともに、体重のコントロールも求められる。ナトリウム・グルコース共輸送体2(SGLT2)阻害剤は、腎臓近位尿細管において糖の再吸収に関与するトランスポーターのSGLT2を阻害し、尿中への糖排出を促進することで血糖値を低下させる全く新しい機序の血糖降下薬である[2)]。従来の血糖降下薬が、インスリン分泌の促進あるいはインスリン抵抗性改善を介して、血糖値は低下させるものの、糖は体内で消費されるのに対し、SGLT2阻害薬では糖そのものが体外に消費されないで尿糖として排出される。この特徴的な機序によるものと考えられる軽度の体重減少効果（2～3%）や降圧効果（5mmHg程度）が治験段階でSGLT2阻害薬に認められており、肥満や高血圧合併2型糖尿病患者に対しては他の血糖降下薬に比べより適した薬剤となることが期待される。

Ⅲ．研究計画

1. 目的

今回の試験では、血糖コントロールが不十分である肥満・高血圧合併2型糖尿病患者100例（予定）を対象に、SGLT2阻害剤であるカナグリフロジン100mgを1日1回24週間投与し、投与前、12週、24週後における、本剤の血糖コントロールの指標（HbA1c、空腹時血糖、HOMA-R、HOMA-β）に及ぼす影響と安全性（有害事象の有無）について評価する。さらにカナグリフロジンの生活習慣病パラメータ（血圧、体重、アディポネクチン、中性脂肪、レムナントコレステロール、アポB48)、心筋障害の指標（ナトリウム利尿ペプチド、トロポニン)、腎障害の指標（シスタチンC、アルブミン、L-FABP、NGAL）や血管障害の指標（高感度CRP）の推移についても検討し、本剤の肥満合併2型糖尿病患者に対する適応性を評価する。

２．対象

下記の試験実施施設通院中の 2 型糖尿病患者で以下の選択基準を満たす男女外来患者を対象とする。参加予定症例数 100 例である。

① 来院時空腹時血糖（FPG）≦270mg/dL で 6.5％≦HbA1c＜10％を満たす患者（スルホニルウレア剤使用時には HbA1c≧7.0％）
② BMI が 25kg/㎡以上の患者
③ 食事療法・運動療法のみで薬物治療がされていない患者、またはスルホニルウレア剤・チアゾリジン系薬剤・ビグアナイド系薬剤・DPP4 阻害剤のうち 1 剤が一定の用量で 8 週間以上単独使用されている患者
④ 血圧が 130/80mmHg 以上の患者
⑤ 試験開始時に文書による同意が取得された患者

ただし、以下の項目のいずれかに該当する場合は対象から除外する。また、治療薬による安全性（薬剤の添付文書を参照）を考慮し問題ある症例や、医師が不適当と判断した症例は対象から除外する。
① カナグリフロジンに過敏症の既往歴のある患者
② 重症ケトーシス、糖尿病性昏睡又は前昏睡、1 型糖尿病の患者
③ 血液透析又は腹膜透析を要する患者を含む重度腎機能障害のある患者（推定 GFR＜30）
④ 重度感染症、手術前後、重篤な外傷のある患者
⑤ 尿路・性器感染症の既往のある患者
⑥ 糖質ダイエットを実施している患者
⑦ 妊婦、または妊娠の可能性のある患者
⑧ 授乳中の患者
⑨ 20 歳未満あるいは 75 歳以上の患者
⑩ 観察期開始 3 ヶ月前以内に治験に参加した患者
⑪ 同意取得 3 ヶ月前以内に脳卒中、心筋梗塞を発症した患者
⑫ インスリン治療中または開始予定患者
⑬ 同意取得時に経口血糖降下薬が２種類以上使用されている患者
⑭ 同意取得時の糖尿病既往歴 5 年以上の患者
⑮ 同意取得時に利尿薬が投与されている患者
⑯ その他、担当医師が本試験実施計画書を遵守するのが困難であると判断した患者

３．被験者の同意

（１）研究の登録に先立って主治医は，本試験について文書で説明し，患者から文書による「同意」を取得する。

（２）患者に十分な判断能力の無い場合を含め本人意思が明確でない場合には同意取得はできない。

４．被験薬

SGLT2 阻害剤：カナグリフロジン錠

５．試験方法

（１）治療薬

カナグル錠 100mg

（２）併用可能薬

経口血糖降下薬は、スルホニルウレア剤・チアゾリジン系薬剤・ビグアナイド系薬剤・DPP-4 阻害薬の中から１剤併用可能。なお、試験期間中の用量変更、新規追加は行わない。合併症治療薬は制限しないが、試験期間中、可能な限り薬剤の用量変更、新規追加は行わない。なお、これらの薬剤と併用を行った場合には、薬剤名、用法・用量を記録することとする。

（３）併用禁止薬

インスリン

（４）方法

選択基準を満たし除外基準に抵触しないことを確認後、文書による同意を取得し、添付文書に記載されている用法・用量に従って、患者にカナグル錠 100mg を１日１回 24 週間経口投与し、投与前、12 週、24 週後に空腹時採血と採尿を実施する。

（５）登録方法のながれ

① 患者来院

② インフォームドコンセント(IC)の取得

③ 投与前の空腹時採血と採尿を実施

（６）資料と試料の移送方法

藤田保健衛生大学医学部臨床検査科に提出する症例報告や検体は、被験者識別コードを用いて連結可能匿名化して郵送する。藤田保健衛生大学医学部臨床検査科より検査を外部委託する場合には、被験者識別コードを用いて連結可能匿名化して郵送する。対応表は外部委託業者には送らない。

6. 検査項目

投与開始前、開始 12 週後、開始 24 週後に血圧と体重測定を行う。同時に空腹時採血と採尿を実施し、以下の項目を測定する。

保険診療内検査	血液検査	ALT, AST, γ-GT, Na, K, Cl, BUN, クレアチニン, 尿酸, LDL-C, HDL-C, TG, HbA1c, 血糖
	尿検査	尿蛋白, 尿糖
保険診療外検査（*）	血液検査	インスリン, 1.5AG, 高感度 CRP, アポ B48、レムナントコレステロール, アディポネクチン, シスタチン C, ナトリウム利尿ペプチド, トロポニン
	尿検査	アルブミン, L-FABP, NGAL, クレアチニン

7. 評価項目

① 血糖コントロール指標（HbA1c、空腹時血糖、HOMA-R、HOMA-β）の推移
② 生活習慣病パラメータ（血圧、体重、アディポネクチン、中性脂肪、レムナントコレステロール、アポ B48）の推移
③ 心筋障害の指標（ナトリウム利尿ペプチド、トロポニン）の推移
④ 腎障害の指標（シスタチン C、アルブミン、L-FABP、NGAL）の推移
⑤ 血管障害の指標（高感度 CRP）の推移
⑥ 安全性（自覚症状、臨床検査値異常）

8. 有害事象が発現した場合の処置

有害事象とは、薬剤が投与された被験者に本臨床研究期間中に生じる、あらゆる好ましくない、あるいは意図しない徴候(臨床検査値の異常変動を含む)、事象のことであり、本臨床研究との因果関係の有無は問わない。

重篤な有害事象とは、薬剤が投与された（投与量を問わず）際に生じるあらゆる医療上のできごとのうち、以下の事象を指す。

1) 死亡に至ったもの
2) 生命を脅かすもの
3) 入院または入院・加療期間の延長が必要となったもの
4) 永続的または重大な機能不全に陥ったもの
5) 先天異常を来したもの
6) その他の重大な医学的事象

死亡、生命を脅かす事象、あるいは入院を要する事象とはならなかった場合でも、被験者を危機にさらしたり、上記のいずれかの結果に至らぬよう内科的あるいは外科的処置を要すると医学的に判断される場合は重篤な有害事象とみなされる。

本臨床研究との因果関係の有無にかかわらず、重篤な有害事象が発生した場合には中止を含めた適切な処置を実施し，速やかに「有害事象報告」を研究責任者に報告する、研究責任者は速やかに、疫学・臨床研究等倫理審査委員会および当該研究機関の長に報告する。
有害事象の簡潔な記述、重篤であると判断した理由、被験者識別コード、研究責任医師、臨床研究名および投与開始日、因果関係（薬剤及び臨床研究手順）の判定を記載するものとする。
なお、必要に応じ、副作用報告書を作成し、当該医薬品の製造元へ連絡するものとする。また、本臨床研究に関連する予期しない重篤な有害事象が発生した場合には、対応の状況と結果を公表し、厚生労働大臣等に報告するものとする。

9. 中止・脱落

有効性が認められない場合や、有害事象あるいは臨床検査値異常変動により継続が困難な場合、症状の悪化の為に継続が困難な場合など主治医の判断による「中止」および患者側の意思もしくは事由による「脱落」についてはその発生日を記録する。また不来院については出来る限り健在などの確認を行う。

10. 実施事項における倫理的配慮

a 実施事項の対象となる者に理解を求め、同意を得る方法

被験者には本試験の概要について文書による説明を行い、被験者の自由意志に基づき参加すること、同意しない場合にも不利益は発生しないこと、いつでも同意を撤回できることを十分に説明の上、文書にて同意を得ることとする。

b 実施によって生じる個人への利益、不利益及び危険性

カナグリフロジンは血糖降下だけでなく体重減少や降圧効果を期待できる。カナグリフロジンの副作用発現率は29.1%（474例/1629例）であった。その主なものは無自覚性低血糖（6.8%）、低血糖症（4.8%）、頻尿（3.4%）、血中ケトン体増加（2.9%）、便秘（2.2%）、口渇（1.6%）、外陰部膣カンジダ症（1.5%）、膀胱炎（1.2%）、ケトーシス（1.2%）などである。その他の本試験実施により予想される危険性として、これらの測定による採血量の増加、採血時の苦痛、静脈炎のリスクの増加が考えられる。そのため、これら不利益および危険性については、事前に文書にて十分に説明し理解いただくとともに、採血時には有害事象を予防するよう細心の注意を払うこととする。また、本試験に関わる費用は、保険適応外の項目については臨床検査科の研究費及び田辺三菱製薬株式会社の資金提供により賄い、被験者に費用負担が発生しないようにする。また、本研

究の実施に起因して被験者に何らかの健康被害が発生した場合は迅速かつ適切な治療を行う。その場合は保険診療で対応する。謝金は予定していない。

c　個人情報の保護方法、試料等の保存と廃棄

個人情報（プライバシー）は、厳重に保護する。研究実施中を含む期間における個人情報の管理責任者は研究責任者とする。データは連結可能匿名化したファイルを用い、元ファイルは医学部 1 号館 302 号室の施錠された保管庫で保管する。方法は、紙の資料の場合は匿名化して保存し、コンピューターデータは USB フラッシュメモリにデータを保存し、パスワードをかけた上、データの暗号化、データの連結可能匿名化を行う。医学部 1 号館 302 号室の施錠可能なロッカーに保管する。キーファイル（対応表）は暗号化し、パスワードをかけ施錠可能な別のロッカーに保管し、研究目的以外には使用しない。パスワードを知る者は研究責任者のみに限定する。キーファイルはインターネットと接続されたコンピュータでは開かない。

結果を学会報告する場合もデータとして発表し、個人が特定できる形では発表しない。またこの試験で得られた結果は、学会、医学専門誌への発表を予定しているが、その際にも被験者の名前など被験者を特定できる情報を含まれないように十分に配慮する。なお、試験の目的以外に、試験で得られた被験者のデータを使用しないこととする。被験者の検体等を病院外に出して測定等を行う必要がある場合は、連結可能匿名化した後の資料番号をもとに行い、個人を特定できない形で測定を行う。なお、被験者の検体は、医学部 1 号館 302 号室で施錠のもとで厳重に保管し、試験終了後は、検体を感染性廃棄物として、蓋のある容器に入れ容器ごと施設の手順に従い廃棄することとする。

試験実施施設で試験実施に係る生データ類及び同意書を取り扱う際は、被験者の秘密保護に十分に配慮する。試験実施施設の個人情報の管理責任者はそれぞれの施設での分担責任者とする。すなわち、平光ハートクリニックでは平光伸也、いしぐろクリニックでは石黒良明、植村循環器科・内科では植村晃久、内山クリニックでは内山達司、可児医院では可児　篤、加藤医院では加藤千雄、加藤内科クリニックでは加藤久視、木村内科では木村　央、徳田クリニックでは徳田　衛、名古屋循環器科・内科では吉田　哲、野場医院では野場万司、長谷川内科では長谷川和生、松原クリニックでは松原史朗、松原内科医院では松原由朗、松山医院では松山裕宇、宮城島内科では宮城島賢二、森内科クリニックでは森　一真、やまだクリニックでは山田健二とする。

藤田保健衛生大学医学部臨床検査科に提出する症例報告や検体は、被験者識別コードを用いて連結可能匿名化して郵送する。連結可能匿名化の対照票（キーファイル）は 2 個作成し、一部は実施施設の施錠可能なロッカーに保管する。もう一部は研究責任者に手渡しで届ける。なお、藤田保健衛生大学医学部臨床検査科

より検査を外部委託する場合には、被験者識別コードを用いて連結可能匿名化して郵送する。連結可能匿名化の対照票は外部委託先には送らない。

本研究に関するデータは研究期間の終了とともにすべてを廃棄するか連結不可能匿名化を行う。本研究で収集した試料は研究終了後に試料番号などを注意深く消去したのちに、破棄する。

d 研究資金、謝金、研究に関わる利益相反等

本研究に関わる資金は田辺三菱製薬との契約に基づく研究費と、臨床検査科研究費により賄うこととする。また、被験者に対する謝金は予定していない。なお，資金提供者である田辺三菱製薬は、本試験に関して、適正使用のための情報提供には関与するが、計画、実施、結果の解析と解釈に関与することはない。また，研究責任者と田辺三菱製薬との間に、利益相反関係に問題がない。

e 遵守すべき諸規則等

「ヘルシンキ宣言」（世界医師会）および「臨床研究に関する倫理指針」（厚生労働省）を遵守する。

f 倫理審査委員会の審査

本研究実施に先立って、研究実施計画書や同意説明文書等の資料内容が、倫理審査委員会で科学的、倫理的に審査され、承認後に実施される。

g 監査

倫理審査委員会や規制当局等による監査が実施される場合は、これを受け入れる。

11. 試験実施計画書の変更等に関する手順

試験計画書の変更の必要性が生じた場合、研究責任者と分担研究者で協議し決定する。

12. 試験実施計画書の不遵守発生時の処置

本試験は ITT の理念に従って評価される（ITT：Intention to treat)。

13. 目標症例数

目標解析症例数 100 例(変動する評価項目を探索的に見出すために必要と考えられる症例数)

14. 実施期間

倫理審査委員会承認後～平成31年9月10日

（症例登録期間　倫理審査委員会承認後～平成27年12月31日）

15. 本臨床研究の登録

研究責任者は，本研究開始前に、UMIN臨床試験登録システムに登録する。

16. データ解析・マネージメント

データ固定前に別途解析計画を作成する．各評価項目については，適切な統計量をもって検討する．有害事象・副作用については，発現例数，件数を算出する．

責任者：藤田保健衛生大学医療科学部准教授　松井　茂

17. 研究責任者

藤田保健衛生大学医学部 臨床検査科教授 石井潤一

事務局

藤田保健衛生大学医学部 臨床検査科准教授 成瀬寛之

〒 470-1192　愛知県豊明市沓掛町田楽ヶ窪 1-98

TEL：0562-93-2312　 FAX：0562-93-2315

18. 試験実施施設（予定）別紙

19. 検査測定機関

1）SRL第1・第2八王子ラボラトリー

〒192-8535

東京都八王子市小宮町51

2）LSIメディエンス中央総合ラボラトリー

〒174-8555　板橋区志村3-30-1

以上

別紙　試験実施施設（予定）

1)	平光ハートクリニック	（名古屋市南区）
2)	いしぐろクリニック	（名古屋市北区）
3)	植村循環器科・内科	（豊明市）
4)	内山クリニック	（知多市）
5)	可児医院	（名古屋市瑞穂区）
6)	加藤医院	（名古屋市西区）
7)	加藤内科クリニック	（名古屋市東区）
8)	木村内科	（犬山市）
9)	徳田クリニック	（名古屋市中村区）
10)	名古屋循環器科・内科	（名古屋市中村区）
11)	野場医院	（豊田市）
12)	長谷川医院	（名古屋市守山区）
13)	松原クリニック	（春日井市）
14)	松原内科医院	（名古屋市南区）
15)	松山医院	（名古屋市緑区）
16)	宮城島内科医院	（静岡市葵区）
17)	森内科クリニック	（岐阜市）
18)	やまだクリニック	（名古屋市中区）
