# Supplementary material for: Possible Increase in Serum FABP4 Level Despite Adiposity Reduction by Canagliflozin, an SGLT2 Inhibitor
Source: PLoS One. 2016 Apr 28;11(4):e0154482. doi: 10.1371/journal.pone.0154482 (PMC4849662; doi:10.1371/journal.pone.0154482)
Supplement: S2 Protocol — (DOCX) [file pone.0154482.s003.docx]

***Trial Protocol***

**Elucidation of parameters of life-style disease and possible biomarkers in relation to cardiovascular and renal disturbance in patients with type 2 diabetes mellitus**

A person in charge:

Junnichi Ishii

Department of Joint Research Laboratory of Clinical Medicine, Fujita Health University School of Medicine, Toyoake 1-98, Aichi, Japan

Edit:

September 10, 2014 (Ver 1.0)

October 7, 2014 (Ver 1.1)

**Contents**

I. Summary of study plan

II. Background of study plan

III. Study plan

1. Purpose

2. Study subjects

3. Consent

4. Drug

5. Methods

6. Items of measurement

7. Assessment

8. Treatment in adverse events

9. Cancellation and withdrawal

10. Ethical consideration

11. Methods of plan change

12. Treatment of disobedience

13. Target number of cases

14. Implementation

15. Registration

16. Data analysis

17. A person in charge and office

18. Institutions

19. Measurement institution

I. Summary of study plan

To investigate the effects of canagliflozin, a sodium-glucose cotransporter 2 (SGLT2) inhibitor, on parameters of life-style disease and biomarkers in relation to cardiovascular and renal disturbance in patients with type 2 diabetes mellitus, who had insufficient blood glucose control, obesity and hypertension.

II. Background of study plan

In patients with type 2 diabetes mellitus and obesity, not only blood glucose control but also body weight reduction is important. SGLT2 inhibitors have a mechanistic drug for diabetes, possible decrease in blood glucose and body weight as well as lowering blood pressure.

III. Study plan

1. Purpose

To investigate the effects of 100 mg/ day canagliflozin, a sodium-glucose cotransporter 2 (SGLT2) inhibitor, for 12-24 weeks on parameters of life-style disease (blood pressure, body weight, etc) and biomarkers in relation to cardiovascular and renal disturbance (BNP, cystatin, L-FABP, FABP, etc) in 100 patients with type 2 diabetes mellitus, who had insufficient blood glucose control, obesity and hypertension.

2. Study subjects

100 patients with type 2 diabetes mellitus as a study plan

Including criteria

1) Fasting plasma glucose ≤ 270 mg/dl and 6.5 ≤ HbA1c <10%

(if sulfonylureas are used, HbA1c ≥ 7.0%)

2) BMI ≥ 25 kg/m^2^

3) No medication with anti-diabetic drugs or only 1 drug among for sulfonlyureas, thiazolidiones, biguanides, DPP-4 inhibitors more than 8 weeks

4) Blood pressure ≥ 130/80

5) Written informed consent was received.

Exclusion criteria

1) Allergy for canagliflozin

2) Severe ketosis, diabetic coma, or type 1 diabetes mellitus

3) Severe renal dysfunction including hemodialysis

4) Severe infection, perioperative period or trauma

5) Past history of urinary infection or genital infection

6) On low carbohydrate diet

7) Pregnancy or possible pregnancy

8) On lactation

9) Age <20 or ≥ 75

10) Clinical trials in the past 3 months

11) Stroke or myocardial infarction in the past 3 months

12) Treatment with insulin

13) More than 2 anti-diabetic drugs

14) More than 5 years of diabetes mellitus

15) Treatment with diuretics

16) Doctor judgment of difficulty of plan procedure

3. Consent

1) Written informed consent is needed to be received.

2) If patients do not have ability of judgment, informed consent can not be received.

4. Drug

SGLT2 inhibitor, canagliflozin

5. Methods

1) Canaguru tab 100 mg

2) no medication or only 1 drug among for sulfonlyureas, thiazolidiones, biguanides, DPP-4 inhibitors more than 8 weeks

3) No treatment with insulin

4) 100 mg once a day for 24 weeks. Collect blood and urine before and after 12 and 24 weeks.

5) Flowchart: coming to hospital, informed consent and collecting blood and urine.

6) Case reports and samples are sent to Department of Joint Research Laboratory of Clinical Medicine, Fujita Health University School of Medicine, after anonymous.

6. Items of measurement

At 0, 12 and 24 weeks. Measurement of blood pressure and body weight. Collection of fasting blood and urine.

ALT, AST, g-GT, Na, K, Cl, BUN, Cr, uric acid, LDL, HDL, triglyceride and glucose

Urinary protein, urinary sugar, etc

Insulin, 1,5AG, high-sensitive CRP, Apo B48, RLP, adiponcetin, cystain c, BNO, toropoin, FABP4, etc

Urinary albumin, L-FABP, NGAL, Cr, etc

7. Assessment

1) Glucose control: time course of HbA1c, fasting glucose, HOMA-R and HOMA-b.

2) Lifestyle disease parameters: time course of blood pressure, body weight, adiponectin, FABP4, triglyceride, RLP and Apo48

3) Cardiac parameters: time course of BNP andtroponin

4) Renal parameters: time course of cysytatin C, albumin, L-FABP and NGAL

5) Vascular dysfunction marker: time course of hsCRP

6) Safety: symptoms and abnormalities of measurement items

8. Treatment in adverse events

Adverse events should be informed to institution and ethic committee.

1) Dead

2) Life-threatening

3) Admission

4) Severe dysfunction

5) Congenital anomaly

6) Others

9. Cancellation and withdrawal

Date of cancellation and withdrawal should be noted.

10. Ethical consideration

a) Written informed consent is received.

b) Benefit and Demerit

Not only lowering blood glucose but also body weight reduction and lowering blood pressure would be expected by the treatment with canaglifrozin. Reported side effects (29.1%: 474/1629 cases) include asymptomatic hypoglycemia (6.8%), hypoglycemia (4.8%), polyuria (3.4%), increased ketone body (2.9%), constipation (2.2%), thirsty (1.6%), candida infection (1.5%), cystitis (1.2%) and ketosis (1.2%). For treatment of side effects, cost would be free.

c) Privacy, preservation and abolition of samples

Privacy is strictly protected. Data are anonymously saved at rocked room. After completion of the study, data files and samples would be abolished.

d) Cost and COI

No honorarium for patients.

Investigators have no COI with Tanabe Mitsubishi company.

e) The study conforms to the principles outlined in the Declaration of Helsinki.

f) The study is performed with the approval of the Ethical Committee of Fujita Health University. Written informed consent was received from all of the study subjects.

g) Audit would be performed by request.

11. Methods of plan change

When needed, investigators are going to make a discussion and decision of plan change.

12. Treatment of disobedience

The study is assessed by ITT (intention to treat).

13. Target number of cases

~100 patients as expected.

14. Implementation

After approvement of ethical committee, till September 10, 2019.

15. Registration

Before beginning the study, the study will be registered in UMIN system.

16. Data analysis

Before completion of data, plan of analysis will be made.

17. A person in charge and office

Junnichi Ishii, Professor

Department of Joint Research Laboratory of Clinical Medicine, Fujita Health University School of Medicine

Office

Hiroyuki Naruse, Associate Professor

Department of Joint Research Laboratory of Clinical Medicine, Fujita Health University School of Medicine, Toyoake 1-98, Aichi, Japan

Tel: 0562-93-2312, Fax: 0562-93-2315

18. Institutions

1) Hiramistu Heart Clinic

2) Ishiguro Clnic

3) Uemura Clinic

4) Utiyama Clinic

5) Kaji Clinic

6) Kato Clinic

7) Kato Naika Clinic

8) Kimura Clinic

9) Tokuda Clinic

10) Nagoya Cardiovascular Clinic

11) Noba Clinic

12) Hasegawa Clinic

13) Matsubara Clinic

14) Matsubara Naika Clinic

15) Matsuyama Clinic

16) Miyagishima Clinic

17) Mori Clinic

18) Yamada Clinic

19. Measurement institutions

1) SRL Hachioji laboratory

Hachioji Komiya 51, Tokyo 192-8535

2) LSI Mediance central laboratory

Itabashi Shimura 3-30-1, Tokyo 174-8555
